# Nanocellulose Materials: Processing, Properties, and Application

**DOI:** 10.3390/nano16070435

**Published:** 2026-04-01

**Authors:** Anthony Burchett, Niccole Callahan, Trey Casini, Aidan De Los Reyes, James Dornhoefer, Subin Antony Jose, Pradeep L. Menezes

**Affiliations:** Department of Mechanical Engineering, University of Nevada-Reno, Reno, NV 89557, USA; aburchett@unr.edu (A.B.); ncallahan@unr.edu (N.C.); dcasini@unr.edu (T.C.); adelosreyes@unr.edu (A.D.L.R.); jdornhoefer@unr.edu (J.D.); subinj@unr.edu (S.A.J.)

**Keywords:** nanocellulose materials, cellulose nanocrystals, cellulose nanofibrils, bacterial nanocellulose, sustainable composites

## Abstract

Nanocellulose materials (CNMs), encompassing cellulose nanocrystals (CNCs), cellulose nanofibrils (CNFs), and bacterial nanocellulose (BNC), have emerged as a versatile and sustainable class of bio-based nanomaterials with significant promise for applications in mechanical engineering. This review systematically examines the processing of nanocellulose via mechanical, chemical, and enzymatic routes, alongside surface modification strategies that enhance performance and address scalability challenges. A principal advantage of CNMs lies in their exceptional mechanical properties, including superior strength, stiffness, and toughness, which position them as high-performance, sustainable reinforcement agents for advanced composites. Beyond mechanical reinforcement, CNMs exhibit a suite of functional properties critical for engineering design, such as thermal stability, tunable conductivity, effective gas/moisture barrier performance, and improved tribological behavior. These characteristics enable their use in diverse high-value applications, including lightweight composites, protective coatings, energy storage devices, sensors, actuators, and intelligent material systems. Furthermore, the inherent renewability, biodegradability, and recyclability of nanocellulose align closely with the principles of a circular economy and green engineering. However, the successful integration of CNMs into mainstream manufacturing requires overcoming key challenges. These include the energy intensity of certain production processes, inherent moisture sensitivity, long-term stability under operational conditions, and compatibility with established industrial techniques. Life-cycle analyses reveal important environmental trade-offs that must be navigated. Overall, nanocellulose represents a renewable, multi-functional material platform whose unique combination of mechanical performance, functional versatility, and environmental benefits is poised to drive innovation in next-generation engineering materials.

## 1. Introduction

Nanocellulose is derived from cellulose, a natural polymer with a deep history of human use. Its applications span from ancient technologies, such as the cordage of 4500 BC and Egyptian papyrus [1], to the modern production of paper. This long-standing utility highlights the enduring importance of cellulose, which contemporary science is now deconstructing to create advanced nanomaterials. While humanity has utilized cellulose for millennia, only recent advances in nanoscience have enabled the deconstruction of its hierarchical structure to unlock its full potential. Cellulose is a naturally occurring linear polymer of β-(1→4) linked *D*-glucose units, forming the primary structural component of plant cell walls, where it provides mechanical rigidity [2].

These glucose chains assemble into the repeating disaccharide unit cellobiose (C_6_H_10_O_5_)*n* and further bundle into parallel, hydrogen-bonded microfibrils, creating the inherent strength and stiffness of the raw material [3].

Nanocellulose is extracted from this native architecture via processing of organic sources such as wood pulp, cotton, or agricultural residues [4]. CNCs can also be isolated from marine biomass such as algae (e.g., Sargassum species), further expanding the range of renewable feedstocks [5]. This isolation yields nanomaterials prized for their exceptional specific strength, providing a high performance-to-weight ratio. For instance, composites reinforced with nanocellulose can demonstrate remarkable toughness. One study reported an impact strength of 4.96 kJ/m^2^ in cotton denim reinforced with nanocellulose [6]. It is this combination of lightweight character and mechanical reinforcement that makes nanocellulose a compelling material for advanced applications.

Nanocellulose is commonly categorized into three primary types: cellulose nanocrystals (CNCs), cellulose nanofibrils (CNFs), and bacterial nanocellulose (BNC) [7]. However, recent advances in nanotechnology have led to the emergence of additional morphologies and hybrid nanocellulose structures, extending beyond this conventional classification [8,9]. These forms (hereafter referred to by their abbreviations) possess unique property profiles and are produced via distinct pathways. CNCs are most commonly isolated from plant cellulose by selective removal of amorphous regions (often via acid hydrolysis), yielding highly crystalline, rod-like particles. In contrast, CNFs are produced by liberating the fibrous network through intensive mechanical shearing, or grinding, resulting in longer, flexible fibrils containing both crystalline and amorphous domains. In addition, CNFs can also be produced through enzymatic pretreatment combined with mechanical fibrillation, which can reduce energy consumption [10]. BNC synthesis is fundamentally different: it is biosynthesized by specific bacterial strains (e.g., Komagataeibacter xylinus) in a controlled culture, requiring optimized nutrients, pH, and temperature to produce a pure, highly crystalline nanofibrillar network [11]. The distinct characteristics of these types enable specialized applications, from reinforcement in composites to unique cultural heritage preservation (for example, BNC has been used in restoring ancient Chinese Xuan paper [12]).

The production of nanocellulose is achieved through three core methods, namely chemical, enzymatic, and mechanical processing, each yielding different material forms. Acid hydrolysis, typically using sulfuric acid, selectively degrades the amorphous regions of cellulose fibers to isolate highly crystalline CNCs. A significant drawback of this method is the generation of acidic wastewater requiring careful remediation. Enzymatic hydrolysis offers a greener alternative, employing specific cellulase enzymes to break down cellulose into nanoscale components, albeit often at a slower reaction rate. Mechanical processes such as high-pressure homogenization or grinding apply intense shear forces to fibrillate biomass, separating it into networks of CNFs while largely preserving the native fiber structure [13].

**Figure 1:**
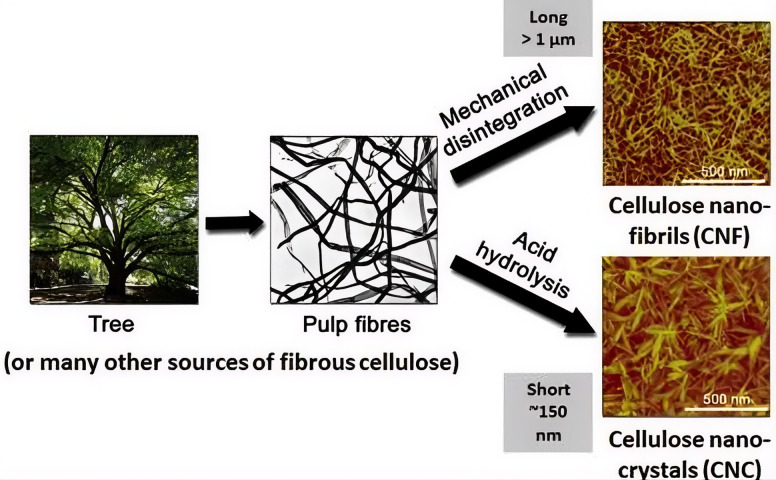
CNC and CNF microstructure. Reproduced from [14], open access, Molecules, MDPI.

In contrast to these top-down extraction methods, bacterial nanocellulose is produced via a bottom-up biosynthesis. Cultivating strains of bacteria (typically Gluconacetobacter or Komagataeibacter) in an optimized aqueous medium leads to the extracellular secretion of pure cellulose nanofibrils. Parameters like nutrient composition, oxygenation, and incubation conditions must be precisely controlled to yield robust BNC pellicles. The resulting BNC exhibits high crystallinity, purity, and often superior mechanical properties [11]. Figure 1 illustrates the fundamental morphological differences between CNCs and CNFs obtained through these various routes.

In addition to conventional chemical and enzymatic routes, several advanced mechanical processes have been developed for the efficient production of CNFs. These include high-pressure homogenization (HPH), grinding, and cryocrushing. In HPH, a cellulose slurry is subjected to extreme pressures (50–2000 MPa) and forced through a narrow valve, imposing intense shear, cavitation, and rapid pressure drops that fibrillate the material. HPH is highly effective but energy-intensive. Grinding employs a disk refiner or stone mill where cellulose pulp passes between static and rotating disks, peeling apart fiber walls into individual fibrils; this is often combined with enzymatic or chemical pretreatments to reduce energy demand. Alternatively, cryocrushing involves freezing water-saturated cellulose (e.g., in liquid nitrogen) to embrittle the fibers, then crushing the frozen mass to create microfractures; upon thawing, these fibers readily disintegrate into a nanofibrillar network [15].

The inherent biological origin of nanocellulose confers two of its most valuable characteristics: biodegradability and biocompatibility. These underpin a diverse application spectrum [2]. In biomedical engineering, nanocellulose’s low toxicity and natural abundance make it an attractive platform for medical devices and therapies. Specific applications include advanced wound care: nanocellulose-based bandages and dressings can provide protective, breathable, and hemostatic barriers. Its high surface area and tunable chemistry also enable use in drug delivery systems—incorporating nanocellulose into hydrogels, aerogels, or particulate carriers can significantly enhance the controlled release and bioavailability of therapeutics [11].

For mechanical engineering, nanocellulose presents a compelling proposition as a sustainable, high-performance material. Its exceptional specific strength and stiffness offer an outstanding strength-to-weight ratio, enabling the design of lightweight yet durable components. As an abundant and renewable nanomaterial, it can be integrated as a reinforcing agent in polymer matrices to create “green” composites that reduce environmental impact compared to conventional synthetic reinforcements [16]. The combination of these mechanical properties with functional versatility and a benign environmental profile positions nanocellulose as a key constituent in the next generation of sustainable engineering materials.

The potential of nanocellulose is significant, yet its path to widespread commercial adoption is not without obstacles. Key challenges include ensuring consistent quality and performance on an industrial scale, as well as competing with well-established, cost-effective synthetic materials in the marketplace. However, nanocellulose possesses a decisive and growing advantage: its sustainable profile. Derived from abundant, renewable biomass, it offers a compelling mix of high performance, biodegradability, and a low environmental footprint, which are attributes increasingly prioritized in modern engineering design. The following sections elaborate on these points, detailing how nanocellulose can overcome existing barriers to fulfill its remarkable potential in mechanical engineering.

## 2. Types and Structure of Nanocellulose

This section details the three primary categories of nanocellulose materials: CNCs, CNFs, and BNC. While CNCs and CNFs are derived mainly from plant sources, BNC is biosynthesized by bacteria, resulting in distinct structural and property profiles. Research interest in all forms of nanocellulose, particularly BNC, has grown significantly in recent years, as evidenced by publication trends. Table 1 illustrates this surge, highlighting a markedly increasing focus on bacterial cellulose compared to plant-derived nanocellulose over five years.

### 2.1. Cellulose Nanocrystals, Cellulose Nanofibrils, and Bacterial Nanocellulose

Cellulose is a ubiquitous natural biopolymer and fundamental building block of the plant kingdom, historically used in applications ranging from paper and textiles to construction materials [18]. Its abundance, renewability, and inherent biodegradability establish it as a cornerstone bio-based material aligned with circular economy principles [18]. Recent advancements in nanotechnology have enabled the isolation of cellulose at the nanoscale, giving rise to nanocellulose materials with dramatically enhanced properties that have become a major focus of R&D [18].

Nanocellulose materials are primarily categorized into three types based on their source and production pathway. The two forms extracted from plant biomass are CNCs and CNFs, while BNC is produced via microbial biosynthesis. In addition, nanocellulose can also be derived from marine animal sources (e.g., tunicates), which yield highly crystalline cellulose with distinct properties. The properties of CNCs and CNFs are strongly linked to their biological source and the specific mechanical or chemical extraction conditions used [19]. A critical distinction lies in their production: plant-derived CNCs and CNFs are obtained through top-down breakdown of bulk fibers, whereas BNC is synthesized bottom-up by bacteria (such as Komagataeibacter xylinus), resulting in a material of very high purity and crystallinity [17].

In practice, nanocellulose fibers can be obtained directly from bacterial culture or by processing plant fibers (or even spinning of synthetic cellulose derivatives) [18]. As noted, CNCs and CNFs form the basic building blocks in nanocellulose technology. BNC, discovered more recently, adds a third dimension to this family. The production methods differ. While plant nanocellulose is derived from the top-down mechanical and/or chemical processing of plant fibers, bacterial cellulose is obtained via a bottom-up synthesis [17]. In essence, all three share the same chemical structure (cellulose), but their sizes, shapes, and certain properties differ due to their origin. For example, CNCs extracted from cotton and wood pulp consist of a few laterally bound crystallites with lengths 100–350 nm and widths ~10–20 nm (thickness ~3–5 nm), whereas those extracted from tunicate are much longer and form fewer bundles [19]. Tunicates, like marine invertebrates, represent a non-plant biological source of cellulose. In general, CNCs are rod-like and highly crystalline, whereas CNFs are longer, more entangled fibers with mixed crystallinity. BNC has the same chemical makeup as plant cellulose but a different morphology, often exhibiting greater flexibility and higher hydrophilicity than plant-derived nanocellulose [17].

### 2.2. Structural Characteristics and Comparison

The source of the cellulose and the extraction method have direct effects on the size, shape, and crystallinity of the resulting nanocellulose. As mentioned, CNCs tend to be rod-like, highly crystalline particles, which are often tens to a few hundred nanometers long. CNFs are much longer (often micron-scale lengths) and form entangled networks of fibrils, including both crystalline segments and amorphous regions. CNFs are typically long, entangled fibrillar structures; however, under certain processing conditions, they can also assemble into nanosheet or nanoplatelet morphologies [20]. Meanwhile, BNC, despite having the same chemical structure as plant cellulose, has a unique ribbon-like nanofiber morphology produced by bacteria [17]. BNC’s biosynthetic origin generally gives it finer fibrils and a higher degree of polymerization, translating to greater flexibility and water-holding capacity compared to plant-based nanocellulose [17].

Overall, the structural forms range from the short, rigid whiskers of CNCs to the long, flexible nanofibers of CNFs, and the ultra-pure nanofiber networks of BNC. These differences underpin their differing behaviors in applications—from reinforcing plastics to forming robust, hydrated membranes.

### 2.3. Emerging Nanocellulose Morphologies and Advanced Structures

Recent advances in nanotechnology have expanded the scope of nanocellulose beyond conventional classifications of CNCs, CNFs, and BNCs. In particular, emerging morphologies such as two-dimensional (2D) nanocellulose nanosheets, nanoplatelets, and hierarchical assemblies have achieved increasing research attention due to their unique structural and functional properties [21,22,23].

Studies have shown that CNFs with diameters in the order of a few nanometers can undergo self-assembly into layered nanosheet or nanoplatelet structures under controlled processing conditions. These assemblies, sometimes referred to as nanocellulose “carpets” or films, exhibit enhanced surface area, anisotropic mechanical behavior, and improved barrier properties compared to conventional fibrillar networks. Such morphologies are typically achieved through controlled drying, shear-induced alignment, or surface functionalization strategies [24].

In addition to 2D nanosheet structures, hybrid nanocellulose architectures have also been developed. These include nanocellulose–graphene-layered systems, aerogel-derived sheet-like structures, and aligned nanofibril assemblies. These advanced morphologies enable improved interfacial interactions, enhanced thermal transport pathways, and superior mechanical reinforcement, particularly in applications such as flexible electronics, energy storage devices, and barrier coatings [25,26].

The development of these emerging nanocellulose forms reflects a transition from traditional material classification toward morphology-driven design. This approach enables the tailoring of nanocellulose structures at multiple length scales, thereby expanding their applicability in next-generation multi-functional engineering systems.

## 3. Synthesis and Processing

Cellulose is the world’s most abundant polymer, commonly derived from wood, cotton, algae, fungi, and many other fiber-rich plants [27,28,29]. The production of nanocellulose materials (CNMs) involves processing and refining these organic sources, breaking them down to their fundamental microfibrils and crystalline domains. Importantly, sources for cellulose are not limited to pristine wood or crops; they can include agricultural waste or forest residues [13], making nanocellulose production potentially very sustainable. Cellulose is also produced by a variety of bacteria during fermentation, yielding BNC that has the same chemical formula as plant cellulose [30]. This wide range of renewable sources allows for diverse manufacturing processes. However, cost versus output and process efficiency are critical considerations for CNM production.

### 3.1. Extraction and Initial Processing of Source Materials

**Figure 2:**
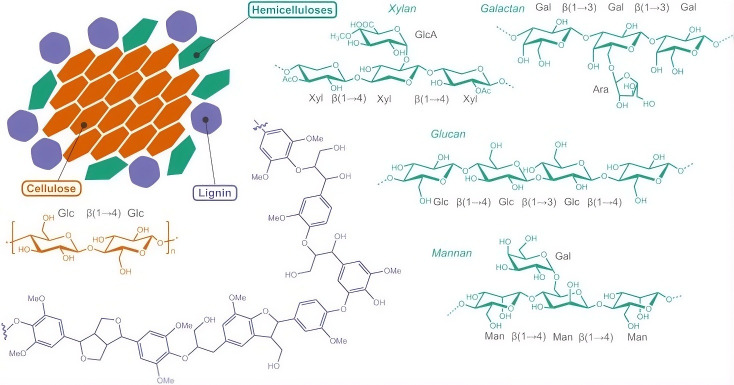
Cellulose macrofibril structure and the molecular structures of its common polymers. Reproduced from [31], open access.

When starting from raw lignocellulosic biomass (e.g., wood, straw, or agricultural waste), the first steps often involve mechanical comminution—grinding, chipping, or pulping the material to increase surface area. This is typically followed by chemical or enzymatic treatments to break down and remove non-cellulosic components (hemicellulose, lignin, pectin, etc.) and isolate cellulose fibers. Lignocellulosic biomass primarily consists of cellulose microfibrils embedded in a matrix of hemicellulose and lignin (Figure 2) [31]. These components form a composite structure (Figure 2) where elementary fibrils ~2–3 nm in diameter aggregate into larger fibril bundles (10–30 nm) surrounded by hemicellulose (bound by hydrogen bonds) and lignin (via covalent bonds) [31]. The exact composition and proportions of cellulose, hemicellulose, and lignin vary by source. Each component has its own useful byproducts, but to produce high-quality nanocellulose, the non-cellulosic fractions typically must be removed or reduced.

The general process flow begins with mechanical preprocessing (grinding, milling, refining, etc.) to break the material into smaller pieces and disrupt the plant cell walls, followed by “pretreatment” steps. These pretreatments can include heat, steam explosion, enzymatic hydrolysis, or chemical treatments (alkaline pulping, organosolv processes, etc.) [28]. The goal is to degrade or solubilize lignin, hemicellulose, pectin, waxes, and other impurities, leaving behind a cellulose-rich pulp [28]. At this stage, cellulose often remains organized in microfibrils. Mechanical shearing (e.g., using high-shear mixers, blenders, or refiners) is then used to break these macrofibrils down into nanoscale fibers. By the end of this sequence, one obtains a suspension of CNFs—a gel or slurry of nanofibrillated cellulose. Further steps like dilution, washing/dialysis to remove residual chemicals, or even electrospinning of the suspension can be applied depending on the desired form of the CNFs [28,29].

### 3.2. Production of Cellulose Nanocrystals

Cellulose nanocrystals are typically produced by further processing cellulose pulp or CNF suspensions to remove amorphous regions and release crystalline rod-like particles. The most common method for producing CNCs is acid hydrolysis. In a typical procedure, a cellulose fiber slurry (often pre-purified) is treated with a strong acid such as sulfuric acid at elevated temperature. The acid preferentially hydrolyzes the less-ordered (amorphous) segments of the cellulose microfibrils, freeing the crystalline segments as individual nanocrystals [32,33]. The process also hydrolyzes some hemicellulose or pectin residues and can partially esterify the CNC surfaces (e.g., sulfate half-ester groups in the case of sulfuric acid hydrolysis). After a controlled reaction time, the mixture is diluted and washed extensively, or dialyzed, to remove acid and reaction byproducts. The resulting suspension contains CNCs—rod-shaped, highly crystalline nanoparticles typically 3–10 nm in diameter and 50–300 nm in length, depending on the source and hydrolysis conditions [32]. Sulfuric acid is most commonly used because it yields negatively charged, colloidally stable CNCs, but other acids (HCl, phosphoric, etc.) can be used to obtain uncharged or differently functionalized CNC surfaces. Yields and properties can vary with different acids. In any case, careful neutralization and removal of acid is required, as well as disposal or recycling of the acidic effluent.

### 3.3. Production of Bacterial Nanocellulose

Bacterial nanocellulose (BNC) is generated through the fermentation of specific bacteria that secrete cellulose nanofibers. The most widely used bacteria are strains of Gluconacetobacter (also known as Acetobacter or reclassified as Komagataeibacter), which naturally produce cellulose as a protective pellicle. To manufacture BNC, these bacteria are cultured in an aqueous nutrient medium containing a carbon source (e.g., glucose), nitrogen source, and other nutrients. Under appropriate conditions (typically static culture in shallow trays, or agitated culture for fibrous forms), the bacteria synthesize and extrude nanoscale ribbons of cellulose that entangle to form a gelatinous mat at the air–liquid interface. After a certain growth period, this cellulose layer can be harvested. The raw BNC must then be purified by removing bacterial cells and byproducts—commonly done by washing and boiling the pellicle in dilute NaOH or other cleaning solutions, then rinsing thoroughly with water [30]. The purified BNC, free of lignin or hemicellulose, consists of an interwoven network of nanofibers (20–100 nm in diameter) with extremely high crystallinity and mechanical strength. BNC is often produced in relatively pure form without the need for harsh chemicals (aside from the base wash), though yields and productivity depend on maintaining optimal culture conditions. Recent research also explores genetic or process modifications to improve BNC production rates and shape (e.g., producing BNC in beads or threads instead of sheets).

### 3.4. Surface Modification and Functionalization

Nanocellulose’s surface is rich in hydroxyl (–OH) groups, which not only contribute to strong hydrogen bonding between fibrils but also provide sites for chemical modification. Surface modification of nanocellulose can impart new functionalities or improve compatibility with other materials. Common approaches include covalent chemical modifications (e.g., esterification, etherification, TEMPO-mediated oxidation), polymer grafting, or adsorption of functional molecules [13]. By tailoring the surface chemistry, one can adjust properties like dispersion in non-polar matrices, thermal stability, or binding affinity for specific molecules.

One widely explored application of surface modification is to create amphiphobic (both hydrophobic and oleophobic) coatings. For example, coating a surface with CNF/CNC and then chemically modifying it (such as grafting fluorinated moieties or performing silylation) can yield a robust, wear-resistant coating that repels water and oils [13]. Such coatings take advantage of cellulose’s high strength and can provide abrasion resistance, impact resistance, and thermal stability, while also being self-cleaning. However, achieving a uniform, well-adhered coating can be challenging because untreated nanocellulose is hydrophilic and tends to agglomerate or have poor wetting on hydrophobic surfaces. This necessitates surface treatments to improve dispersion and bonding. For instance, one can graft polymer chains or small hydrophobic molecules onto the nanocellulose surface to compatibilize it with hydrophobic polymers or solvents [34,35]. Grafting can be done through reactions like isocyanate coupling, or via oxidizing and then coupling functional groups (e.g., periodate oxidation to dialdehydes, followed by reaction with polymers) [36,37]. The strong intra-network hydrogen bonds and high crystallinity of nanocellulose make direct chemical penetration difficult, often requiring activation steps or solvents [38].

In the case of BNC, functionalization can be achieved either in situ (during fermentation, by adding functional additives that get incorporated) or post-synthesis via similar chemical reactions [39]. Functionalizing nanocellulose can enhance interfacial bonding in composites, introduce electrical conductivity, impart antimicrobial properties, etc. For instance, attaching silanes or other coupling agents can improve the bonding between CNFs and a polymer matrix [40]. The key is to alter the surface enough to get the desired interaction, but without degrading the cellulose backbone or compromising its mechanical integrity.

### 3.5. Challenges of Nanocellulose Production

Despite many advantages, producing nanocellulose at large scale faces notable challenges. Because cellulose is a structural biopolymer, it is naturally resistant to many solvents and enzymes (a trait known as biomass recalcitrance) [29]. This means that breaking it down into nanoform often requires intense processes. For example, cellulose does not melt or thermally process easily; it undergoes pyrolysis before melting, so it cannot be directly melt-spun like some polymers, complicating certain fiber production techniques [29]. Many CNM production methods are labor- and energy-intensive, involving multiple steps that are not easily automated. High-pressure homogenization, for instance, consumes significant energy per kilogram of output. In addition, nanocellulose production often yields suspensions with low solid content (to avoid gelation), meaning subsequent drying or thickening steps are needed, which can cause issues like hornification (irreversible agglomeration upon drying).

All these factors lead to high input costs and moderate yields. The processes can be slow and require careful control. Therefore, current research aims to streamline nanocellulose extraction by combining steps, using enzymes to reduce mechanical energy, recycling reagents, or developing new technologies (e.g., flow-through reactors, continuous reactors for acid hydrolysis, twin-screw extrusion approaches, etc.). Scalability and efficiency improvements are crucial to make nanocellulose cost-competitive with synthetic materials.

## 4. Mechanical Properties

Cellulose, as a critical load-bearing component in plant cell walls, has intrinsically high crystallinity and thus a notably high Young’s modulus and tensile strength (higher than that of cast iron on a per-weight basis) [13]. When reduced to a nanoscale, these cellulose-based fibers retain impressive mechanical properties, often surpassing synthetic fibers. For example, the strength-to-weight ratio of nanocellulose can be up to eight times that of stainless steel [13,41]. Nanocellulose materials can form dense, self-assembled structures via hydrogen bonding and van der Waals forces, contributing to their mechanical robustness and also to notable thermal stability [41]. Moreover, being a natural polymer, cellulose is biodegradable, which is increasingly relevant for material life-cycle considerations. It is also worth noting that all forms of nanocellulose (CNC, CNF, BNC) are inherently transparent when sufficiently dispersed, which can be advantageous for optical applications.

### 4.1. Physical Properties of CNFs, CNCs, and BNC

Each form of nanocellulose exhibits impressive mechanical qualities, though there are differences. CNFs (10–100 nm diameter fibrils with alternating crystalline and amorphous segments) have slightly lower stiffness and strength compared to CNCs or BNC due to their amorphous content, but they are very flexible and tough. CNCs, being highly crystalline rods about 3–10 nm in width and a few hundred nanometers long, display higher rigidity. Individual CNCs have been reported to have an axial Young’s modulus on the order of 100–160 GPa (comparable to aluminum) and tensile strength around 7–10 GPa [32]. Typical bulk measurements for CNC films show a Young’s modulus of 20–30 GPa and tensile strength of ~300 MPa, but these can vary with processing. BNC’s fibrils are larger in diameter (20–100 nm), and BNC mats often have even higher crystallinity than CNCs extracted by acid, which can translate to high tensile strength as well. Some BNC pellicles have tensile strengths on par with or slightly above those of CNC films, though BNC’s properties are very sensitive to cultivation conditions [42]. An outstanding feature of CNFs and BNC (more so than CNC) is their high water content and porosity; BNC in particular can hold a large amount of water, which is useful for certain applications (e.g., wound dressings) but can reduce measured strength when wet.

### 4.2. Effect of Source Material and Processing on Properties

The mechanical performance of nanocellulose is influenced by the cellulose source and the processing parameters used for extraction. For example, cellulose from higher plants (woody biomass) often has higher intrinsic tensile strength than that from lower plants (like annual crops) due to differences in microfibril angle and cellulose content [43]. The crystallinity index of the cellulose (fraction of crystalline regions) depends on the biomass source and affects stiffness and biodegradability—the higher the crystallinity, generally the stiffer and slower to degrade [31]. Processing conditions such as reaction times, temperatures, and mechanical energy input can also alter nanocellulose properties. Harsher conditions might increase crystallinity (by further removing amorphous parts) but could shorten the fibers and introduce defects, affecting strength.

When produced optimally from high-quality sources, nanocellulose can achieve remarkable mechanical benchmarks: for instance, a stiffness exceeding that of Kevlar and tensile strength beyond cast iron (on a density-adjusted basis) has been cited for well-prepared CNMs [43]. In the case of BNC, although many bacteria can produce cellulose, only certain strains produce it fast and efficiently enough for scale [30]. BNC’s properties are highly affected by factors like nutrient ratios, oxygen supply, incubation time, and reactor design (static vs. agitated cultures) [42]. These influence fiber diameter, degree of polymerization, and branching of the BNC network, which in turn affect the mechanical performance of the final BNC material.

## 5. Functional Properties Relevant to Mechanical Engineers

Nanocellulose carries many advantages for engineering beyond strength alone. In particular, its functional properties, thermal behavior, barrier performance, tribological (friction and wear) characteristics, etc., complement its mechanical strength and make it viable for a wide range of applications. Recent research emphasizes how nanocellulose’s tunable nanoscale architecture and surface chemistry enable a balance of performance and sustainability. As one review notes, nanocellulose exhibits exceptional mechanical properties (low density, high flexibility, and strength while being chemically inert) [16]. Additionally, these materials offer superior routes of functionalization, which are efficacious, economical, and sustainable [44]. Together, these qualities demonstrate why nanocellulose is attracting global attention as a next-generation material. In the subsections below, we highlight several key functional traits of nanocellulose that are particularly important for mechanical engineering applications: thermal stability and conductivity, gas/moisture barrier properties, and tribological performance.

### 5.1. Thermal Stability and Conductivity

The thermal properties of nanocellulose-based materials can be modified through processing and structural alignment. Pure cellulose nanofibril networks, in their native state, have low thermal conductivity (since cellulose is an insulator). However, surface modifications or composite strategies can significantly enhance thermal performance. For example, coating CNFs with a thin polydopamine layer increased the thermal conductivity of a CNF film by nearly 50%, by improving interfibril heat transfer [45]. This result underscores the importance of interfacial engineering for thermal management. Another study reviewed by Gan et al. [46] showed that incorporating nanocellulose into polymer composites generally improves thermal stability and can alter the degradation behavior, effectively allowing more controlled thermal performance than the base polymer alone.

In engineering design, materials often need to operate across a range of temperatures. If a material has low thermal stability, its use is limited to niche conditions. Fortunately, nanocellulose’s thermal behavior can be customized. In some cases (such as heat-spreading components in electronics), higher thermal conductivity is desired; in others (like insulation), low conductivity is preferred. Sato et al. [47] highlighted that among the crystalline forms of cellulose, the cellulose Iβ polymorph exhibits higher thermal stability, making it a more suitable candidate for thermal management uses that involve prolonged exposure to heat. Moreover, alignment of nanocellulose can dramatically influence heat transport. One study demonstrated that when nanocellulose fibrils are highly aligned and bonded together as continuous filaments, the material can exhibit surprisingly high thermal conductivity along the fiber direction [48]. This is because phonon transport (heat conduction mechanism) is more efficient along the well-connected, oriented cellulose chains.

The ability to control thermal stability and conductivity through structural alignment and surface treatment makes nanocellulose extremely versatile. It means that the same basic material can be tuned either as a thermal insulator or a thermal conductor. For example, nanocellulose aerogels have been developed as ultralight thermal insulators, achieving thermal conductivities as low as 0.025 W·m^−1^·K^−1^—better than some polyurethane foams and mineral wool insulations. This superior insulation performance arises from the aerogel’s porous architecture, leveraging the Knudsen effect and phonon scattering to minimize heat transfer [49]. Such materials are promising for energy-efficient building insulation and thermal management in electronics. On the other hand, by integrating nanocellulose with conductive fillers (like graphene or graphite), researchers have created composite films with greatly enhanced heat conduction. For instance, nanocellulose–graphene hybrid membranes achieved in-plane thermal conductivity around 8.8 W·m^−1^·K^−1^ (and ~1.13 W·m^−1^·K^−1^ through thickness) [50]. These values are orders of magnitude higher than those of pure nanocellulose, demonstrating that nanocellulose can form the scaffold of high thermal conductivity materials for heat dissipation in electronics.

The adaptability of nanocellulose’s thermal properties is a large advantage. Engineers can select and modify a nanocellulose material based on whether a component needs to insulate or conduct heat. Because both extremes (insulative aerogels and conductive films) can be reached with appropriate modifications, nanocellulose-based solutions can be applied across diverse contexts—from building insulation panels to flexible electronic substrates. An added benefit is that using nanocellulose in these roles introduces biodegradability into domains (like electronics) where end-of-life waste is a growing concern, potentially mitigating e-waste by substituting for non-degradable components.

### 5.2. Barrier Properties (Gas and Moisture)

Nanocellulose materials naturally form dense networks via hydrogen bonding, especially when dried into films. These dense cellulose fiber networks can inhibit the diffusion of gases, making them promising as oxygen or aroma barriers in packaging. Tayeb et al. [51] demonstrated that by hot-pressing CNF films and applying surface coatings, the oxygen transmission rate (OTR) could be reduced from over 500 cm^3^/(m^2^·day) to less than 5 cm^3^/(m^2^·day) even at high humidity. This dramatic improvement shows that processing and surface treatment can yield nanocellulose films with excellent gas barrier properties, suitable for protective packaging and coatings.

However, one important caveat is that nanocellulose’s barrier performance is not uniform for all gases—specifically, nanocellulose is usually much better at blocking oxygen than water vapor. Owing to the hydrophilic nature of cellulose, moisture can penetrate or be absorbed relatively easily. As one study notes, pure nanocellulose has a high resistance to oxygen permeation but poor water vapor barrier properties due to the hydrophilic nature of cellulose [52]. In practical terms, this means nanocellulose films can keep out oxygen (and certain other gases) effectively, which is great for preventing oxidation of food or sensitive products, but they struggle to keep out moisture unless modified. Future successful implementations of nanocellulose-based barrier films will likely have to capitalize on cellulose’s inherent strengths while compensating for its weaknesses [53]. For example, combining nanocellulose layers with thin hydrophobic coatings or laminating nanocellulose with other biopolymers could achieve a balance—taking advantage of cellulose’s oxygen barrier and strength, and mitigating its moisture sensitivity.

In summary, the barrier properties of nanocellulose highlight its potential in sustainable packaging. Dense CNF or CNC films are essentially breathable to water vapor but nearly impermeable to oxygen under dry conditions. Researchers are optimistic that with further development (such as hybrid structures or chemical modifications), nanocellulose films can meet real-world requirements for packaging, replacing petroleum-based plastics in some uses. The combination of renewability, strength, and tunability (via coatings or composite layers) makes nanocellulose a focus of continued research for sustainable barrier materials.

Indeed, recent advances in chemical modification have shown that nanocellulose films can be made much more hydrophobic. For instance, silylation treatments using vapor-phase methyltrichlorosilane have been applied to regenerated cellulose films, yielding a material with exceptionally low oxygen and water vapor transmission rates, while still maintaining high optical transparency (~87%) and a tensile strength of 146 MPa [54]. Achieving such dual barrier performance (against both oxygen and moisture) in one step is a significant breakthrough, suggesting that nanocellulose-based films can compete with multilayer conventional packaging. Materials that maintain strong barrier properties with minimal processing steps offer cost and manufacturing advantages, and they make nanocellulose films increasingly competitive with traditional packaging films that often require multiple layers or coatings to achieve similar results.

### 5.3. Tribological Performance (Friction and Wear)

Nanocellulose materials can enhance the friction and wear behavior of composites and lubricants. Incorporating even small amounts of nanocellulose into polymers or rubbing interfaces has shown to reduce wear and sometimes lower friction. For example, Shi et al. found that adding CNCs into a poly (methyl methacrylate) (PMMA) matrix decreased wear volume by up to 90%, as well as reduced the friction coefficient, compared to neat PMMA. In another study, Okubo et al. [55] tested bulk CNF material sliding against steel and observed identifiable improvements in friction and wear under both dry and lubricated conditions, indicating that nanocellulose itself can act as a low-friction, wear-resistant bulk material. These findings point to potential uses of nanocellulose in tribological applications—such as filler in wear-resistant composites or even as a bulk-bearing material in low-load scenarios.

Supporting evidence comes from studies on nanocellulose as an additive in lubricants. One report noted that cellulose nanoparticles showed noticeable friction-reducing and anti-wear properties within certain concentration ranges and base oil compositions, and that CNCs and CNFs performed about equally well in this regard [56]. In that case, friction reduction on the order of a few percent was achieved, which, in lubrication engineering, can be significant for energy savings and component life. Another study demonstrated that adding CNCs to water-based lubricants drastically reduced wear: at 500 rpm rotational speed, the wear volume was cut by 99%, and at a low speed (63 rpm) the coefficient of friction was halved [57]. These dramatic improvements suggest that nanocellulose could serve as an effective anti-wear additive, possibly forming protective films on the surface or acting as tiny ball bearings at the interface.

Reducing wear is key to extending the life of mechanical components. Since nanocellulose can provide such benefits while also being environmentally benign (especially compared to some metallic or mineral oil additives), it is an attractive emerging technology in tribology. As a natural, potentially biodegradable additive, it aligns with the trend of making lubricants more eco-friendly (so-called “ecolubricants”).

Further investigations have revealed interesting mechanisms when nanocellulose is used in tribology. For instance, testing of pure CNF molding against steel surfaces (with a lubricant) yielded ultra-low friction coefficients between 0.01 and 0.02. This is a drastic reduction compared to typical sliding materials. The underlying mechanism was attributed to tribochemical reactions: the hydroxyl groups on cellulose react with fatty acids in the lubricant, effectively hydrophobizing the cellulose surface and preventing the usual hydrogen bonding that contributes to friction. Essentially, a thin, lubricating film forms in situ on the cellulose, allowing it to slide with minimal resistance. This finding is remarkable because it shows that cellulose can be a core ingredient in “superlubricity” systems.

Moreover, biodegradable grease formulations containing microfibrillated cellulose (MFC) have been shown to perform well. Greases with 1–7% MFC exhibit gel-like rheology but still flow at temperatures as low as –50 °C, addressing cold-weather lubrication needs [58]. The friction and wear reduction in such systems operates via two distinct pathways. At low cellulose concentrations, the tiny fibrils fill in surface asperities, smoothing the contact. At higher concentrations, the fibrils help build a protective tribofilm on the surfaces. This dual mechanism means engineers can adjust the nanocellulose content to optimize performance for a given application—lower amounts for smoothing, higher for film-forming protection.

In summary, nanocellulose’s tribological potential extends from bulk self-lubricating composites to lubricant additives and surface coatings. It can significantly reduce friction and wear, which is crucial for energy efficiency and durability in mechanical systems. Additionally, using a biodegradable, renewable additive in lubricants could reduce environmental impact (e.g., in case of leaks or disposal). This makes nanocellulose an exciting prospect not just in mechanical engineering materials, but also in the maintenance and sustainability of machinery.

## 6. Applications in Mechanical Engineering

Nanocellulose materials offer a variety of promising applications in mechanical engineering and related fields. Some near-term applications include the following: nanocellulose-reinforced composites for lightweight, high-strength structures; wear-resistant coatings and films for packaging or protective layers; components in energy storage devices (e.g., supercapacitor electrodes, battery separators); and elements of multi-functional sensors, actuators, and smart materials. Below, we discuss each of these areas.

### 6.1. Nanocellulose-Reinforced Composites

Incorporating nanocellulose into composite materials can create lighter and stronger composites, but it also introduces new manufacturing considerations. For example, one study showed that adding a low fraction (~1–5 vol%) of CNFs into a polypropylene (PP) matrix increased the tensile modulus by up to 30% in a material intended for additive manufacturing. However, this reinforcement also increased the melt viscosity, which led to a reduced deposition rate in the 3D printing process [59]. This means manufacturers face a trade-off: improved stiffness versus more challenging processing. Solutions include modifying the printing process or the material preparation (such as using pre-dried, low-moisture nanocellulose and optimized extrusion conditions) to mitigate the viscosity and moisture issues.

A recent review article detailed the technological advances enabling CNFs to serve as viable reinforcements in lightweight structural composites [11]. Traditional fiber reinforcements like glass and carbon fiber, while providing strength, are limited by higher density, high energy costs of production, and poor recyclability. CNFs emerge as a sustainable alternative due to their bio-based origin and high specific strength. Their nanoscale dimensions can increase toughness by hindering crack propagation in the composite matrix. As nanocellulose technology has matured, research has branched into myriad composite systems that exploit these properties.

Studies have demonstrated that even low loadings of CNCs or CNFs can significantly improve the mechanical properties of polymers if they are well-dispersed and bonded. For instance, Missale et al. [60] and others found that adding a few weight percent of CNC/CNF to polymer films can increase tensile modulus and toughness simultaneously, provided there is good interfacial bonding between the nanocellulose and matrix. These results indicate that a small amount of lightweight nanocellulose can promote efficient load transfer within the composite, enhancing stiffness without a heavy weight penalty.

**Figure 3:**
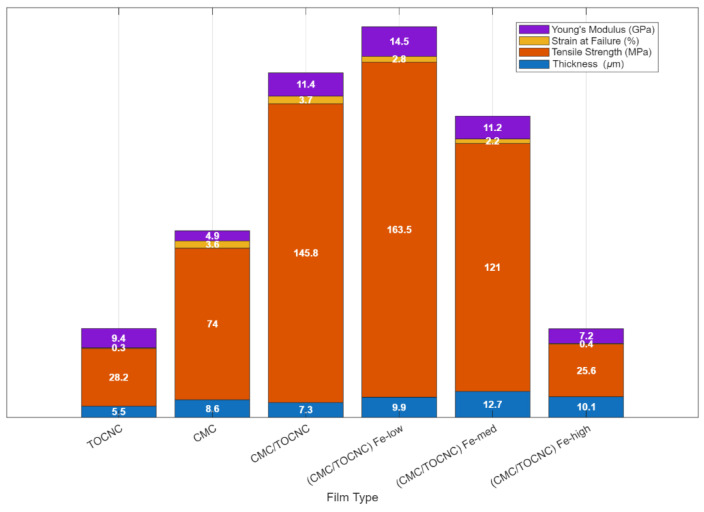
Mechanical properties of different films. Reproduced from [60], open access.

It is important, in these systems, to ensure the nanocellulose is well-dispersed and does not agglomerate, as agglomeration would create weak points. Rheology and processing must be managed carefully because nanocellulose’s tendency to form networks can make the resin more viscous. In the case of Missale et al.’s [60] work, they specifically examined TEMPO-oxidized CNC (TOCNC) used alone versus as a reinforcement in a carboxymethylcellulose (CMC) matrix. Pure TOCNC film was very stiff but also brittle; when a small amount of TOCNC was embedded in CMC, the composite films showed simultaneous increases in strength, Young’s modulus, and toughness (shown in Figure 3). The TOCNC provided reinforcement while the CMC maintained ductility, achieving a combination of properties that is typically hard to get in one material (high stiffness and toughness together). This underscores how nanocellulose enables a composite to have a better balance of mechanical properties.

Beyond traditional composites, CNFs are being applied in manufacturing techniques like 3D printing. Hwang and Gardner [59] reported using spray-dried CNFs mixed into PP as a feed for melt extrusion 3D printing, achieving notable stiffness improvements at various CNF loadings. They found that CNFs could be processed in filament form and actually helped alleviate some feeding issues (the CNFs reduced problems of polymer dripping or inconsistent flow, possibly by increasing melting strength). The main trade-off observed was that higher CNF content increased viscosity, making extrusion and printing more difficult. Their recommendation was to use moderate CNF content and ensure the material is well-dried and thermally stable during printing to avoid degradation. This exemplifies how nanocellulose can be integrated into advanced fabrication processes, provided the material formulation is tuned for the process.

Nanocellulose paper-like materials (pure CNF or CNC “papers”) have also been studied as a form of composite. For example, the mechanical properties of dense CNF papers depend strongly on the microstructure: better nanofiber alignment and careful drying (to reduce porosity) yield higher stiffness and strength [61]. The addition of other nanoparticles to such papers (to introduce electrical conductivity, for example) can adversely affect mechanical properties, as those particles can act as stress concentrators and reduce the paper’s ductility and toughness. Studies also show that environmental conditions (humidity, temperature cycling) can degrade CNF paper properties over time, so protective coatings or hybrid structures are needed to ensure longevity [61]. This research, conducted in the early 2020s, set the stage for integrating CNFs into manufacturing, highlighting the need to control microstructure and the environment for durable performance.

In summary, nanocellulose-reinforced composites open pathways to lighter, stronger materials, but they require careful engineering of processing methods. The hydrophilicity and high surface area of nanocellulose mean that controlling moisture and dispersion is critical. The research so far is very promising—significant mechanical enhancements at low filler content—but industry adoption will depend on overcoming processing challenges (like viscosity management and drying) on a large scale.

### 6.2. Coatings and Films for Wear Resistance and Packaging

Nanocellulose can be cast or coated into films that serve as wear-resistant coatings or barrier layers, for instance, in packaging. De Lima et al. [62] conducted detailed studies showing that CNF/CNC mixtures can be cast into thick, self-supporting films with excellent tensile strength and even some oxygen barrier capability. By hydrophobizing the surface or blending with small amounts of polymer binders, the water resistance and wear resistance of these films can be improved, making them suitable as protective coatings for packaging applications. In other words, nanocellulose films can protect surfaces from abrasion and moisture to a degree, while also being bio-based.

Specific implementations include electroplated CNF-metal coatings and CNC-reinforced polymer coatings, both of which have shown marginal improvements in wear resistance in testing [62]. These coatings leverage nanocellulose to either increase hardness or to form a tight fiber network in the coating, which helps resist wear. The improvements might not always be dramatic compared to high-performance synthetic coatings, but as the formulations and methods are refined, such coatings could become competitive, especially where sustainability is a priority.

Durability under cyclic loading is another concern for mechanical components (bearings, joints, panels, etc.). In one study, nanocellulose-reinforced paper composites were subjected to cyclic humidity and temperature loading to simulate long-term use. They exhibited a ~20–30% drop in stiffness after 1000 cycles, whereas comparable carbon–fiber composites dropped only ~10% under the same conditions. The greater performance loss in nanocellulose composites was attributed to moisture build-up in cellulose’s nanoscale pores during cycling, which plasticized the material [63]. This outcome reinforces the need for reliable moisture barriers or surface treatments when using nanocellulose in environments with fluctuating humidity. Thin nanocellulose-based coatings themselves can serve as barrier layers. For example, Herrera et al. [64] studied plasticized and cross-linked nanocellulose coatings on paper and found significant improvements in barrier properties: oxygen permeability as low as 0.7 mL·μm/(m^2^·day·kPa) at ~50% relative humidity, and a 60% reduction in water vapor permeability compared to uncoated paper. The coating also contributed mechanical reinforcement to the paper. This indicates that nanocellulose coatings can effectively protect a substrate, even in semi-humid conditions, provided the nanocellulose is appropriately modified (plasticized or cross-linked to reduce brittleness and moisture sensitivity).

In packaging technology, one vision is to use a very thin layer of nanocellulose as a coating on conventional paper or bioplastic, to impart an oxygen barrier and a bit of strength, and then apply an ultra-thin hydrophobic layer (which might also be derived from bio-sources) to handle moisture. This could replace multilayer plastic films that currently dominate food packaging.

### 6.3. Energy Applications

Nanocellulose is also finding roles in energy-related applications. Due to its high surface area and porosity (especially CNFs and BNC), it can be an excellent scaffold for electrodes in energy storage devices like supercapacitors or batteries. Choudhary et al. [50] and Wang et al. [65] detail efforts using nanocellulose to create porous, high-surface-area electrodes and separators. In one approach, a porous nanocellulose scaffold was coated with graphene to produce a nanocomposite electrode with improved capacitance [65]. The nanocellulose provided a lightweight, electrically insulating framework that could hold conductive graphene in a large 3D network, thus combining high surface area for charge storage with a robust structure.

**Figure 4:**
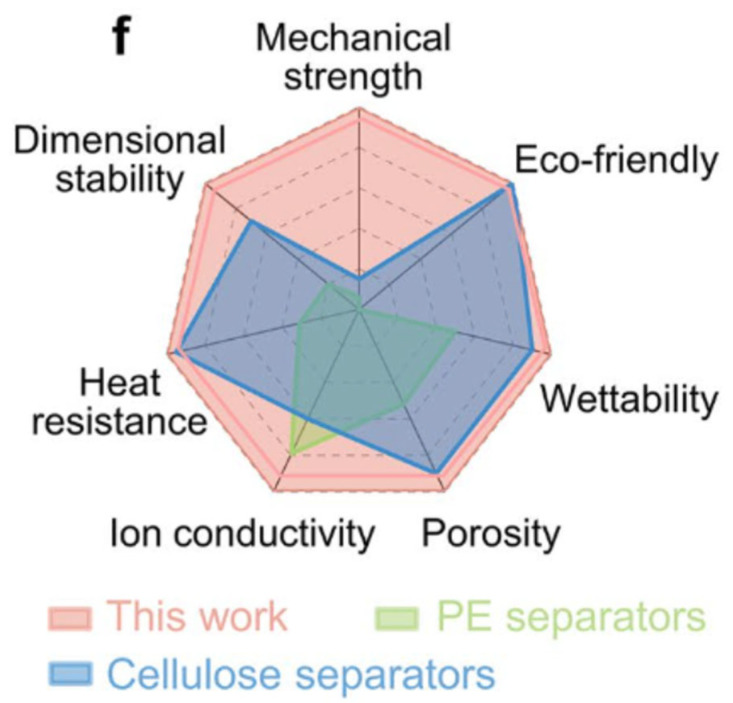
Heat map comparing the SORC film with other separators. Reproduced from [66], open access.

Wang et al. [66] presented a cellulose-based separator for supercapacitors that contains a reassembled nano-cracked film structure to improve electrolyte wettability. This separator, called SORC (solvent-reassembled cellulose) film, had tiny cracks or channels at the nanoscale, which allow electrolyte ions to penetrate more easily while still maintaining the separator’s role (preventing electrical shorting between electrodes). The result was improved ionic conductivity and overall better supercapacitor performance. A comparative analysis (illustrated by a radar chart in Figure 4) showed the SORC separator outperforming conventional cellulose and polyethylene (PP) separators in multiple metrics, from thermal stability to environmental friendliness. This suggests that nanocellulose-based separators can offer a compelling combination of safety (cellulose chars rather than melts, so it can be safer in overheating scenarios), mechanical stability, and sustainability.

Other researchers (Turrosi, Zhao, etc.) have corroborated these findings with cellulose-based membrane separators that provide good mechanical strength and thermal stability compared to PP separators [66,67]. They also note that cellulose separators can have safer failure modes (they do not shrink or melt closed like PP does, which can be a positive safety feature). On the downside, some of these advanced separators require additional processing or additives (like integrating graphene or ceramic particles for certain properties), which can reduce the overall environmental advantage if not done carefully (for example, adding processing steps might increase energy use and complexity).

Yet another energy application is in electrodes for all-solid-state supercapacitors (ASSCs). One study layered CNFs with MXene sheets (MXenes are 2D transition metal carbides [68,69,70]) to prevent the MXene sheets from restacking, thereby maintaining ion pathways in a solid-state supercapacitor electrode. The CNFs acted as spacers and binders, leading to a device that achieved a volumetric capacitance of 94.2 F/cm^3^ and retained ~98% of its initial capacitance after 10,000 charge/discharge cycles. This endurance indicates that the mechanical resilience afforded by the CNFs (preventing electrode structure collapse or densification during cycling) made the device very stable. It reflects the broader trend: nanocellulose can improve the mechanical integrity and longevity of energy storage components.

Thus, nanocellulose contributes to the energy sector by enabling greener, often better-performing components like separators and electrodes. Its advantages include chemical tunability (it can be made to hold electrolyte or be ion-conductive via functionalization), thermal stability, and formability into various structures (aerogels, membranes, films). These properties complement the needs of advanced batteries and supercapacitors.

### 6.4. Sensors, Actuators, and Smart Materials

Nanocellulose’s high surface area, flexibility, and ability to form percolating networks make it suitable for novel sensor and actuator systems, especially in combination with conductive or responsive additives. Singh et al. [71] and Liu et al. [72] have reviewed the use of CNCs and CNFs in various sensor applications. The surface chemistry of CNCs (many –OH groups and easy modification) allows immobilization of chemical recognition elements, making them good platforms for chemical or biological sensors. The percolated network of a CNF film, especially when combined with conductive nanoparticles (like silver nanowires or carbon nanotubes), can form a piezoresistive or capacitive sensor—essentially a strain sensor that changes electrical resistance or capacitance when stretched. Because nanocellulose networks can deform and then recover, they can serve as the scaffold in flexible, wearable sensors that measure motion or strain.

**Figure 5:**
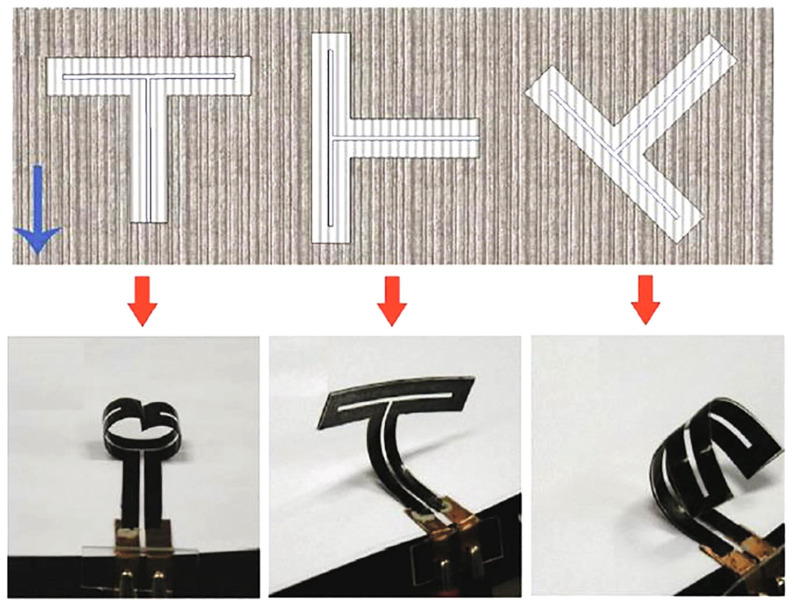
Nanocellulose-based actuator bends when applied with a voltage. Reproduced from [74], open access.

For soft actuators (e.g., in soft robotics), the challenge is to find materials that bend or change shape in response to stimuli like electric fields, while being lightweight and possibly biocompatible. Researchers have developed electroactive nanocellulose-based hydrogels where CNCs or CNFs are combined with ionic liquids or conductive polymers. In such a composite, applying an electric field causes ions to migrate and the hydrogel to bend (electro-osmotic or iono-elastic actuation). Reid and Hamad [73] demonstrated an electroactive actuator using CNCs in a hydrogel, which bent in response to an electric field. Figure 5 shows an example of a cellulose-based bending actuator (essentially artificial muscles) under an applied voltage.

Nanocellulose’s role in such systems is often as a reinforcing, high-surface scaffold that can hold other functional components (like ionic liquids, conductive nanoparticles, or responsive polymers) in a stable architecture. The CNC or CNF network prevents excessive swelling and gives mechanical strength to the soft material, while the other components provide actuation or sensing functionality.

One can envision embedding these nanocellulose-based sensors or actuators into structural composites. For example, a carbon fiber composite could be layered with a thin CNF-based strain sensor. Because nanocellulose is lightweight and minimally invasive, it could be integrated without significantly affecting the composite’s mechanical performance, yet it would endow the structure with sensing capability (detecting strain, impacts, etc.). Similarly, a nanocellulose-based actuator could be part of a smart material system that responds to electrical input to adjust stiffness or shape.

A key consideration for these applications is ensuring the nanocellulose elements remain durable under load. If a nanocellulose sensor inside a composite cracks or delaminates when the composite is stressed, it might lose functionality. Therefore, interface engineering—perhaps grafting polymers onto nanocellulose to better bond with a matrix, or encapsulating the nanocellulose sensor in a protective layer—can be necessary. Multi-scale modeling is also useful to predict how these embedded systems will behave.

Despite these challenges, the prospects are exciting: nanocellulose could allow for “smart” composites that are not only strong and light but also capable of self-monitoring (sensing damage or load) or even self-adjusting (via actuators). Given the increasing demand for intelligent materials in aerospace, robotics, and biomedical devices, nanocellulose’s contribution here could be quite impactful.

In summary, whether it is serving as the backbone of a flexible sensor or as the matrix of an ionic actuator, nanocellulose greatly broadens the functional scope of materials available to mechanical engineers. It brings the benefits of flexibility, low weight, and compatibility with a range of functional dopants, all while being derived from renewable sources. Any given application of nanocellulose comes with its benefits and drawbacks. Nanocellulose offers a lightweight strategy for enhancing structural stability, improved wear and moisture resistance through coatings and blending, better conductivity or ion transport in energy devices, and new possibilities for sensors and actuators. However, the techniques to process and optimize nanocellulose for each application often add cost or complexity. Some industries have been slow to adopt nanocellulose because, despite its performance advantages, the cost-to-performance ratio currently cannot beat cheaper, traditional materials in certain cases. Continued innovation in processing and a greater emphasis on sustainability may tip this balance, making nanocellulose integration more economically attractive in the near future.

## 7. Sustainability and Environmental Impact

With rising concerns about plastic pollution and climate impact, the sustainability of new materials is a crucial consideration. Nanocellulose stands out for being bio-derived and (in most cases) biodegradable. However, producing and using nanocellulose in a truly sustainable way requires careful attention to the environmental impacts at each stage. In this section, we explore nanocellulose’s biodegradability, recyclability, and life-cycle environmental profile, as well as its role in the circular economy and green engineering.

Increasing interest in biodegradable alternatives to plastics has driven research into nanocellulose composites, especially in combination with other biodegradable polymers. For instance, Aigaje et al. [75] reviewed various nanocellulose–biopolymer systems (like nanocellulose with starch, chitosan, PLA, PVA), emphasizing their sustainable properties. Almost every process to manufacture functional CNC/CNF for the applications discussed above has some environmental impact (energy use, chemical use, etc.), which draws attention to the sustainability of the whole process, not just the material itself. Regulations in many countries are now pushing manufacturers to reduce carbon footprints and enhance product recyclability. The positive aspect is that most nanocellulose materials are inherently biodegradable (unless irreversibly combined with non-degradable components). Major producers are therefore trying to use nanocellulose in ways that ensure the final product remains biodegradable or at least more recyclable than conventional composites. On the other hand, many nanocellulose production methods can consume a lot of energy (for mechanical fibrillation, drying, etc.), so there is a need to optimize these processes or supply them with renewable energy to truly claim environmental benefits.

### 7.1. Biodegradability and Recyclability

Multiple studies show that cellulose-based nanomaterials (CNC, CNF, BNC) are biodegradable under the right conditions (presence of cellulolytic microbes, moisture, time). The rate of biodegradation depends on factors like crystallinity and the presence of other substances. When nanocellulose is embedded in a non-biodegradable polymer (e.g., conventional PP plastic), the composite might not biodegrade at all, thereby negating one of nanocellulose’s key advantages. Therefore, recyclability and design for disassembly become important. Patsnp Eureka [76] and others have discussed how to design nanocellulose composites for easier end-of-life processing, for example, by using water-soluble binders or reversible crosslinks. One concept is an “all-cellulose” composite, where both matrix and reinforcement are cellulose-based, so that the entire material can be recycled in a single stream or even re-dissolved and reused. A specific example is the all-cellulose ionic conductive composite (ACICC) described by Copenhaver et al. [77], which can enter a circular bioeconomy loop. In that scheme, the nanocellulose composite membrane can be easily separated, reprocessed, and reused, demonstrating a closed-loop cycle for a biomaterial. Another type of biopolymer being more commonly used is cellulose acetate (CA), finding application in packaging and textile industries, as well as eyeglasses, which contributes the most to CA waste [78].

An Oak Ridge National Lab report [77] investigated how adding CNFs affects the recyclability of common thermoplastics like PLA (polylactic acid) and PETG. They found that composites with cellulose nanofibrils retained mechanical strength better over multiple recycling (re-melting and molding) cycles than the polymer alone. Specifically, after a couple of reprocessing cycles, the CNF-filled PLA kept more than 80% of its original tensile strength, whereas neat PLA fell below 60%. The CNFs likely act as reinforcement that does not break down as quickly under heat, and they may also retard polymer chain degradation by absorbing some thermal stress. Additionally, CNFs seemed to slow the loss of molecular weight in the polymer by perhaps scavenging radicals or just by reducing the mobility of the polymer chains during processing. These findings suggest that incorporating nanocellulose not only makes a material stronger initially but can help it stay stronger after being recycled, thus encouraging recyclability.

Another advantage of nanocellulose noted in coatings research is that even if a product is not biodegradable, it might be more recyclable. For example, in a high-performance coating with CNF, if the coating needs to be removed or recycled, it might be easier to do so than for a complex petrochemical coating [79]. However, nanocellulose’s hydrophilicity is a double-edged sword: as mentioned earlier, moisture can degrade performance over time. For recyclability, this means if a product gets wet repeatedly or is used outdoors, the nanocellulose could weaken, complicating recycling (because the material might fail or fragment before it can be collected). This reiterates the importance of designing protective strategies for nanocellulose in use.

In summary, from a biodegradability and recyclability perspective, nanocellulose offers significant benefits, but you get the most out of it when it is paired with similarly green materials or when designed to be extracted at end-of-life. Composites purely made of biodegradable ingredients (cellulose, starch, PLA, etc.) will likely be compostable or at least digestible by microorganisms. Composites of nanocellulose with non-degradable plastics might need to be recycled mechanically. The field is moving towards systems where nanocellulose helps polymers be reused more times or where the nanocellulose itself can be reclaimed and reused in new products.

### 7.2. Life-Cycle Assessment Perspective

A major promise of nanocellulose is that it could reduce the overall environmental footprint of materials by replacing high-impact constituents like fiberglass or carbon fiber with a renewable alternative. To verify this, life-cycle assessment (LCA) studies have been conducted. One review of CNF production LCA found that the global warming potential (GWP) of CNF (per kg) could range from 0.5 to 3 kg CO_2_-equivalent, depending on the production method and energy source used [80]. For context, these values are in the same order as many common plastics, and lower than aluminum or carbon fiber production per kg. However, if a particular chemical pretreatment like carboxymethylation is used, the GWP can be on the higher end of that range due to additional chemical inputs and processing steps [80]. Enzymatic or purely mechanical pretreatments tended towards lower GWP in that study, but often yield trade-offs like higher electricity usage or lower throughput.

A cradle-to-grave assessment of nanocellulose must consider not only its renewable origin and end-of-life biodegradability, but also the environmental challenges associated with feedstock processing, fibrillation or hydrolysis, washing, drying, transportation, and waste remediation. In particular, mechanical fibrillation techniques can be highly energy-intensive, while acid hydrolysis pathways introduce additional environmental impacts related to chemical consumption, recovery, and wastewater treatment [81]. Thus, the overall carbon footprint of nanocellulose is strongly dependent on the production pathway, energy source, scale of operation, and degree of reagent recycling. Compared with petroleum-based alternatives, nanocellulose can offer clear environmental advantages in renewability, biodegradability, and reduced fossil-resource dependence. However, these benefits are only fully realized when process energy demand and chemical burdens are effectively minimized through optimized manufacturing and supply-chain integration [82,83].

One drawback identified is that scaling lab processes to an industrial scale might raise energy demands significantly. For example, an LCA indicated that an optimized lab-scale CNF process, when scaled up, could require 20–50% more electricity simply due to scale inefficiencies and the need for larger throughput (pumps, mixers, etc., running longer or at higher power) [84]. This suggests that some lab processes do not scale linearly and may need redesign for industrial efficiency.

Kane et al. [85] performed a harmonized LCA/LCI (life-cycle inventory) for CNC and CNF and found that the environmental impacts (GWP, eutrophication, acidification, etc.) depend very strongly on process route, chemicals used, yields achieved, and how drying is handled. For instance, acid hydrolysis routes for CNC can lead to higher GWP and potential toxicity impacts unless the acid is recovered and recycled, because producing and neutralizing acid has a big footprint. Mechanical and enzymatic routes avoid these chemicals but might have a larger electricity burden (especially if run on non-renewable electricity). The study also highlighted a large sensitivity to scale—small batch processes might actually have lower impact per kg in some cases because they bypass the complexities of continuous operation, but generally, economy of scale should improve some metrics if processes are optimized. Another key point was water use: nanocellulose production can use a lot of water (for rinsing, etc.), so water treatment and recycling will be important in large-scale operations to avoid environmental downsides in water consumption or pollution.

In summary, LCA studies show that nanocellulose can be a low-carbon, low-impact material, but it is not automatically so. It heavily depends on how it is made. Feedstock choice matters too: using waste biomass vs. virgin wood might change land-use impact and biodiversity considerations. Energy source is crucial—running a CNF plant on renewable electricity vs. a coal-powered grid makes a big difference in GWP. The use of chemicals like strong acids, alkalis, or organic solvents will add impacts unless a circular process is in place to reuse those inputs. Therefore, engineers and managers looking to use nanocellulose should pay attention to the production pathway of the material they source. If one supplier uses a greener method, the embodied carbon of the nanocellulose could be much lower than another’s. It also means that ongoing research into energy-efficient and chemical-efficient nanocellulose production (like the development of new enzymes or the invention of low-energy mechanical fibrillation techniques) directly translates to making nanocellulose a truly green material at scale.

### 7.3. Role in Circular Economy and Green Engineering

Within a circular economy framework, nanocellulose offers several opportunities. First, it can be derived from waste (agricultural residues, recycled paper, etc.), which gives value to what would otherwise be disposed. This not only provides a feedstock that does not compete with food or lumber, but also helps manage waste. Second, products made from nanocellulose can be designed for reuse or recycling. Unlike thermoset composites or multi-material assemblies that are hard to recycle, nanocellulose composites can, in principle, be made to come apart (for instance, water-dispersible composites) or to be repulped like paper.

Researchers have looked at using nanocellulose to reinforce materials in ways that facilitate repair or remanufacturing. For example, self-healing materials incorporating nanocellulose are being explored [86], which could extend product lifetimes. Using water-based processing for nanocellulose (as opposed to organic solvents) aligns well with green engineering, since water is a benign solvent and easier to recover.

That said, implementing nanocellulose in a circular economy context requires holistic planning. Engineers must consider the following: (1) sustainable sourcing of cellulose (e.g., from fast-growing plants or waste biomass rather than old-growth forests), (2) energy-efficient modifications and minimal use of non-renewable additives, and (3) end-of-life pathways like designing components so that nanocellulose can be separated and composted or recycled. Surface treatments that we apply to nanocellulose to solve technical problems (like moisture sensitivity) often involve adding substances that are not biodegradable (e.g., synthetic polymers, crosslinkers, etc.), which could compromise the circularity of the material. A balance must be struck between immediate performance needs and long-term sustainability.

One vision is that a future low-carbon material economy could use nanocellulose as a ubiquitous reinforcement or scaffold: product designers would use renewable feedstocks, join them in ways that can be undone (for recycling), and ensure any performance-enhancing additives do not prevent material recovery or safe biodegradation. Achieving this will require integrating LCA at the design stage, as well as creative engineering to develop treatments that may be washed off or degraded on command at end-of-life.

In summary, nanocellulose is quite aligned with green engineering ideals—renewable feedstock, compatibility with recycling/biodegradation, and potential to reduce reliance on fossil resources. But to truly deliver on that promise, the entire life cycle needs to be considered. Producing nanocellulose with renewable energy, using it in designs that maximize its benefits (lightweighting, etc.), and planning for its end-of-life will collectively determine how sustainable it really is in practice. There is ongoing research and industrial interest in overcoming current barriers (cost, performance under moisture, etc.) so that nanocellulose can be widely commercialized sustainably. It is important to note that significant R&D is still required to fully commercialize nanocellulose for widespread use in a sustainable way. There are encouraging efforts to keep CNC/CNF biodegradable by using biodegradable matrices or greener pretreatments (like enzymatic processes). The trade-off often comes as higher energy consumption in production stages (drying, mechanical fibrillation), which some users may be reluctant to incur unless regulations or market forces favor sustainable products strongly. Nonetheless, as energy grids become greener and processes improve, nanocellulose’s prospects as an eco-friendly material grow stronger.

## 8. Challenges and Future Directions

While nanocellulose holds great promise, several challenges remain before it can realize its full potential in industry. Key issues include stability (especially regarding moisture), scalability of production, and integration with advanced manufacturing technologies. In this section, we discuss these challenges and outline future directions for research and development.

### 8.1. Stability, Moisture Sensitivity, and Durability

One of the prominent challenges identified for nanocellulose materials is their performance under moist or humid conditions. For example, using nanocellulose in food packaging is desirable due to environmental regulations pushing for biodegradable packaging materials. Bacterial nanocellulose (BNC) is being explored in this context because it is considered ecological, safe, biodegradable, and chemically pure for food contact use [87]. At the same time, researchers are also looking at sustainable feedstocks for nanocellulose to enhance overall green credentials. An interesting approach is using brewer’s spent grain (BSG), a byproduct of the beer brewing industry, as a cellulose source. This medium would repurpose a massive waste product (hundreds of millions of tons globally) into nanocellulose for packaging, making use of something that would otherwise be discarded [88,89,90]. Similarly, fibers from plants like roselle have been studied as viable sources for eco-friendly composites [91]. Nanocellulose (especially CNCs) has even been suggested for use in diverse industries such as food, cosmetics, textiles, and security labeling, underscoring how broadly it could be applied [19].

However, the major hurdle for nanocellulose in packaging is moisture sensitivity. Cellulose-based films, on their own, generally have poor water vapor barrier properties because cellulose readily absorbs water. This means that in humid conditions or with high-moisture contents (such as fresh food), pure nanocellulose packaging would quickly soften or allow moisture transfer, which is unacceptable for many applications. As noted earlier, due to poor barrier properties and high sensitivity to moisture, the application of paper-based food packaging remains limited [92]. To overcome this, various methods are being tried. One example is developing composite or coated materials: a study coated a paper substrate with CNF derived from hemp waste that was chemically modified with a quaternary ammonium compound, which significantly improved the paper’s resistance to moisture and also provided some antibacterial activity [93]. This kind of surface treatment (ammonium-functionalized nanocellulose) can impart hydrophobicity to the inherently hydrophilic cellulose surface.

**Figure 6:**
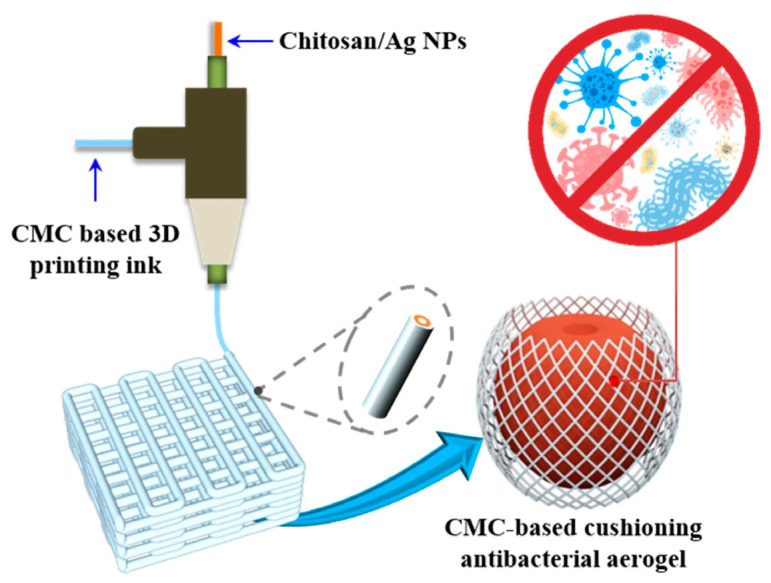
Nanocellulose-based 3D printable food packaging. Reproduced from [94], open access.

So far, these approaches (like using BNC, agricultural waste fibers, and chemical coatings) are in relatively early stages of research but show multiple pathways to tackling the moisture issue. By improving moisture resistance, nanocellulose-based packaging could become viable for a wider range of products, reducing the need for petroleum-based plastics. The drive to find these solutions is strong, given the environmental imperative to replace plastics and the potential utilization of waste streams (like BSG or crop residues) to produce packaging material. In the coming years, we can expect to see further innovations such as blending nanocellulose with biopolymers that are hydrophobic, multilayer structures that include a nanocellulose layer for strength, and an outer hydrophobic layer for moisture, or novel cross-linking techniques that make nanocellulose less water-sensitive. One example of multilayer structures is developments with 3D printing aerogel that combine a hydrophobic surface of carboxymethyl nanocellulose (CMC) with a cushioning silver nanoparticle (AgNP) core [94]. This combination leads to a moisture-repellent aerogel without issues with water retention. Figure 6 shows such 3D-printed food packaging using nanocellulose and AgNPs.

Another aspect of stability is long-term durability which refers to how nanocellulose materials hold up under sustained loads, UV exposure, or over time. Moisture remains the key degradation factor (wet/dry cycling can cause swelling and shrinkage, weakening the structure), but ultraviolet light or certain temperatures can also degrade cellulose (since it is organic, UV can yellow it or break glycosidic bonds slowly). Research into adding UV stabilizers or antioxidants, or chemically modifying nanocellulose to be less prone to UV degradation (e.g., acetylation can sometimes improve UV stability), may be needed for outdoor applications.

In summary, tackling nanocellulose’s stability issues, especially moisture, is critical for its broader adoption. The current trajectory of research suggests a multi-pronged solution: use inherently more moisture-tolerant forms like BNC for some uses, derive cellulose from sustainable sources to keep costs and impacts low, and apply advanced coatings or chemical modifications to impart the needed moisture resistance. Each of these comes with its own challenges (e.g., coatings must be food-safe, modifications must not sacrifice biodegradability), but steady progress is being made.

### 8.2. Scaling up for Industrial Applications

Scaling up nanocellulose production from lab to industry presents two main challenges: process energy requirements and product form handling. A 2020 study highlighted these issues specifically for CNF scale-up [95]. First, the typical methods to produce CNFs—high-pressure homogenization, high-energy ball milling, microfluidization, and ultra-low temperature (cryogenic) crushing—are all extremely energy-intensive. These methods also encourage the cellulose fibrils to form hydrogen bonds with each other (since water is removed or intense forces bring fibrils into contact), leading to hard agglomerates that are difficult to re-disperse [95]. Breaking these agglomerates would require even more energy and could damage the cellulose’s crystal structure. In an industrial setting, running such equipment at scale continuously could be cost-prohibitive unless significant improvements in energy efficiency or throughput are made.

The second factor is that CNFs are usually produced and stored as a dilute slurry (often only a few percent solid content) to prevent them from agglomerating or forming a gel that cannot be handled. Transporting or storing this dilute slurry implies moving a lot of water around, which is inefficient. If one tries to dry the CNFs to reduce volume, they typically form a solid mass that is not easily re-dispersible (hornification). So, producers often ship them as gels or pastes, which limits how far they can economically travel and how they can be incorporated by end-users. One strategy is to produce CNFs on-site or near-site where they will be used (e.g., a paper mill producing CNF to integrate into paper). Another method is to find ways to dry CNFs into a powder that can be rehydrated later without losing properties—research is being done on freeze-drying with additives or spray-drying with certain disintegrating aids to achieve this.

Additionally, scale-up often brings up issues of consistency and quality control. Ensuring each batch of nanocellulose has the same fiber length distribution, surface chemistry, and purity is non-trivial. Industrial processes need robust online monitoring and possibly feedback control to reach consistency (for instance, measuring viscosity or particle size in real time).

To address energy concerns, some newer methods are being researched: using twin-screw extruders to mechanically fibrillate fiber in a somewhat continuous process, which could potentially harness mechanical energy more efficiently; using low-cost renewable electricity for processes (like running a homogenizer at off-peak times when wind power is abundant, etc.); developing enzyme-assisted mechanical processes that allow shorter or less forceful mechanical treatments.

For the handling issue, one approach is to never fully dry the nanocellulose until it is in its final product form. For example, instead of producing dry CNF, a manufacturer could directly produce CNF dispersion and feed it into a composite production line (mixing with resin, etc.). This integration could mean nanocellulose is always handled in wet form, circumventing the need to ship water or break agglomerates.

In the case of CNCs, scale-up has its own challenges: handling large volumes of acid safely and economically, and dealing with acid recovery. Some companies have developed continuous reactors for acid hydrolysis to produce CNCs by pumping pulp and acid through at controlled conditions, then quenching and separating the acid, which can be energy-intensive but is being optimized. Pilot plants exist and are ramping up to multiple tons per day in some places.

In short, scaling nanocellulose production is an active area of process engineering research. The two factors of energy and format (dry vs. wet) are central. If those can be solved—for example, a breakthrough that allows producing CNF at 20% solids as a pumpable gel, or a mechanical process that uses 10x less energy—then nanocellulose could become dramatically more accessible and cost-effective.

From an economic standpoint, nanocellulose is not yet uniformly cost-competitive with commodity materials. Recent market and review sources show that polyethylene and PET are commonly near the low-$1/kg range, and glass fiber is also typically around ~$1–1.5/kg [96,97]. Carbon fiber is much costlier, often in the tens of dollars per kilogram. In comparison, nanocellulose remains more variable because selling prices depend strongly on type, purity, solids content, and application [98]. Recent literature suggests CNC is still commonly priced above commodity polymers, while CNF is more promising for scale-up in bulk applications, and BNC remains the most expensive due to fermentation, purification, and drying costs. Accordingly, full-scale industrial applicability will require higher production volumes, continuous processing, reduced drying and fibrillation energy demand, improved reagent recovery, and tighter process standardization [9].

### 8.3. Integration with Advanced Manufacturing

The use of nanocellulose in modern manufacturing extends beyond replacing or reinforcing conventional materials; it also includes enabling new technologies and products. We have touched on some advanced applications (energy devices, electronics, 3D printing, sensors), but looking forward, integration with cutting-edge manufacturing processes will be crucial.

One area is energy storage and electronics. There is growing interest in using nanocellulose composites for components in batteries and electronics to reduce their environmental impact. Portable electronic devices contribute to global waste and resource use, so replacing some plastic or even metal parts with nanocellulose composites could help. Nanocellulose has very desirable properties for flexible electronics: great mechanical properties, low density, high surface area, and minimal thermal expansion [99]. These make them ideal as substrates or supports in flexible circuits, displays, or wearable devices. Additionally, for large-scale energy storage, nanocellulose-derived carbon materials are being investigated. For example, carbonizing nanocellulose aerogels can produce carbon aerogels for electrodes that have a hierarchical porous structure. Nanocellulose’s structure can template carbon materials that are useful in supercapacitors or batteries [100]. Since nanocellulose can yield high-performance carbon electrodes (with structured porosity and high surface area), it is being looked at for applications like electric vehicle batteries or grid storage systems that require performance and scalability.

Another frontier is 3D printing and additive manufacturing. Nanocellulose has been used to modify rheology of inks or resins for 3D printing, as briefly mentioned. It can also reinforce printed structures. For example, in bioprinting or printing of soft structures, adding nanocellulose can improve the mechanical integrity of the printed object without making it too brittle. Researchers found that nanocellulose can help stabilize conductive hydrogel inks for printing flexible sensors: it gives the hydrogel mechanical strength and prevents it from collapsing during printing, whilst being biocompatible and not interfering with conductivity (especially if it is modified to be more conductive or just used as a minor component). Similarly, for printed electronics, like conductive patterns on paper or plastic, a challenge is that substrates are sensitive to moisture and wear. A coating of hydrophobized nanocellulose (like the “nanoworm” from Lakovaara et al. [101]) can act as a protective layer to make these printed electronic circuits more durable and water-resistant.

There is also interest in self-healing and smart composites. As mentioned, Chiriac et al. [86] developed nano-composites with CNFs and a copolymer that had hydrophobic and self-healing properties. The idea of a self-healing composite is that if it gets a microcrack, the material can autonomously repair that crack (for instance, via remelting a bit or using residual forces to close it). CNFs provide a network that holds things together and might even participate in pulling the material back or providing sites for re-bonding. Self-healing is particularly attractive in coatings and electronics (to heal minor scratches or circuit breaks) and in difficult-to-repair structures (like aerospace components). Nanocellulose could be a key ingredient in developing these self-healing systems, given its flexibility and surface chemistry (which could be engineered to rebond when triggered by heat or moisture).

In advanced reinforced composites, like carbon fiber or glass fiber composites, there is an exploration of using nanocellulose as a secondary reinforcement or interfacial agent. For example, CNCs have been studied as reinforcements in bio-based resins or in aligning along with carbon fibers to potentially improve the composite’s compression strength or shear properties [102]. The unique ability of CNCs to align under magnetic or electric fields could also be used to create oriented reinforcing networks in a composite, which might be turned into a manufacturing step in the future (similar to infusing a nanocellulose suspension into a prepreg and then applying a field to align it before curing).

Another emerging application is water treatment and environmental remediation. CNCs can be functionalized to grab pollutants [103]. Manufacturing-wise, one could integrate nanocellulose into filters or membranes for water purification. For example, making a membrane where CNFs are the matrix and adding specific binding sites for heavy metals could lead to a new class of water filters. These could be processed in sheets or hollow fibers using techniques borrowed from paper-making or electrospinning, indicating a crossover between traditional manufacturing and nanomaterial processing.

Multi-material systems: Mechanical engineering is moving towards multi-functional materials (structural batteries, load-bearing antennas, etc.). Nanocellulose’s multi-functionality (mechanical + electrical/ionic + optical, etc.) makes it a good candidate to tie into these concepts. For instance, one could envision a structural component in a car that is also an energy storage device—nanocellulose could be part of the matrix that stores energy while also contributing stiffness.

Overall, the advanced manufacturing integration of nanocellulose is about leveraging its unique traits in newly engineered systems, rather than just swapping it into existing ones. Future directions likely include scaling production of tailored nanocellulose forms (like very long CNFs, surface-modified CNCs, etc.) for specific industries; developing composite formulations that mix nanocellulose with other nano-additives (like combining nanocellulose with nanoclays, graphene, etc., to get hybrid effects); and ensuring compatibility with emerging manufacturing methods (like robotic deposition, roll-to-roll printed electronics, etc.). The goal is to incorporate nanocellulose in ways that enhance performance and add sustainability without disrupting manufacturing workflows—once that is achieved, industry uptake will accelerate. An additional exciting area is biomedical devices. While not a core mechanical engineering field, it overlaps, e.g., 3D-printed nanocellulose scaffolds for tissue engineering, or nanocellulose in wearable health monitoring devices, etc. Integration with biofabrication (like printing tissues or organs) could see nanocellulose as a component in bioinks to add viscosity and strength, which is something already being looked at. Overall, integrating nanocellulose with advanced manufacturing and technology sectors is a rich field of innovation. It promises to bring sustainability to high-tech areas and add new functionalities (like self-healing and sensing) to structural materials. The challenges lie in ensuring nanocellulose can be produced and delivered in the right form for these uses, and that any barriers (like compatibility or processing constraints) are overcome with clever engineering solutions.

## 9. Conclusions

Nanocellulose is derived from abundant, renewable organic materials and offers a remarkable combination of high mechanical strength and low weight. These inherent properties, alongside biodegradability and biocompatibility, make nanocellulose a highly desirable material across multiple engineering disciplines. In mechanical engineering, in particular, nanocellulose stands out as a tough, lightweight, and sustainable material with significant potential. The three main categories of nanocellulose—CNC, CNF, and BNC—each possess unique structural and mechanical characteristics suited to different applications. CNCs provide rigidity and high crystallinity for reinforcement purposes, CNFs offer flexibility and water absorption capacity ideal for films, coatings, and flexible composites, while BNC, being microbially grown, exhibits exceptional purity and strength useful in biomedical and specialized industrial uses.

Beyond its basic physical advantages, the true value of nanocellulose is its versatility. Through various processing methods, nanocellulose’s form and surface can be tuned so that the material can be incorporated into either flexible or stiff systems as needed. For mechanical engineers, this tunability opens up possibilities in designing reinforced composites with tailored properties. For example, nanocellulose-reinforced polymers have shown improved tensile strength, stiffness, and barrier properties without significant weight gain. Such composites are ideal for applications like automotive panels, aerospace interior components, and lightweight robotic structures, where maximizing strength while minimizing mass is crucial. Moreover, nanocellulose’s high specific surface area and ability to interface with conductive nanomaterials (like carbon nanotubes or graphene) mean it can serve as a sustainable scaffold in high-tech devices such as supercapacitors, flexible batteries, and strain sensors. In these cases, nanocellulose contributes to the performance (by providing a structured, porous support or flexible matrix), while also improving the environmental profile of the device. These innovations exemplify how sustainable materials like nanocellulose can drive advancement in high-performance technologies, merging materials science with mechanical engineering design.

From an environmental standpoint, nanocellulose is more than just an engineering material; it represents a shift toward renewable and circular materials. Unlike petroleum-based plastics which can persist as pollutants for centuries, nanocellulose is capable of natural decomposition, reducing long-term waste and microplastic pollution. This attribute has led to the development of biodegradable nanocellulose-based packaging, coatings, and films that maintain needed mechanical durability (for product protection) without causing environmental harm after disposal. Thus, nanocellulose enables engineers to meet performance requirements and also address sustainability goals, which is a dual benefit unique to bio-derived materials.

However, large-scale production of nanocellulose faces hurdles such as high energy consumption, the need for effective dewatering/drying techniques, and the costs of equipment. Mechanical engineers have a key role in surmounting these limitations by designing and optimizing production systems. Improvements in equipment like homogenizers, refiners, and cryogenic mills can lower energy inputs or increase throughput for nanocellulose production. There is also active work on integrating renewable energy into the production process (for instance, using solar or wind power for running energy-intensive grinders) to ensure that manufacturing nanocellulose is itself sustainable. Another ongoing research thrust is surface modification techniques that make nanocellulose more compatible with hydrophobic polymers (since many industrial plastics are hydrophobic). By improving compatibility and dispersion (for example, via spray-drying CNFs with a bit of carboxylation or grafted polymer), nanocellulose could be more easily adopted in existing plastic processing lines, broadening its industrial usability.

Looking ahead, close collaboration among materials scientists, mechanical engineers, and sustainability experts will be vital to fully realize nanocellulose’s potential. Material scientists can develop new forms of nanocellulose with enhanced properties (like larger aspect ratio CNFs or functionalized CNCs), mechanical engineers can devise structures and processes to implement these materials effectively, and sustainability experts can guide decisions so that environmental benefits are maximized. This multidisciplinary approach will help navigate trade-offs, such as balancing performance gains with the carbon footprint of processing.

Industries such as construction, aerospace, biomedical devices, and packaging are all searching for materials that meet performance requirements while also improving sustainability. The market demand for nanocellulose-based solutions is expected to expand significantly as these industries commit to reducing their environmental impact. For instance, in packaging, nanocellulose coatings and films can extend food shelf life as part of biodegradable wrapping materials, offering eco-friendly alternatives to plastics. In biomedical applications, nanocellulose’s biocompatibility and strength make it ideal for tissue scaffolds and wound dressings, supporting regenerative medicine and patient recovery without introducing harsh synthetic materials. Additionally, the general push to replace conventional plastics in many consumer and industrial products aligns well with nanocellulose innovation: every component or product that switches to a nanocellulose composite or film reduces reliance on fossil fuels and can lead to more sustainable product life cycles.

As research continues and more success stories emerge, nanocellulose is poised to become a cornerstone of sustainable engineering and design. This is especially true in mechanical engineering, where one is constantly balancing performance, efficiency, and environmental impact. Nanocellulose offers a rare combination of high performance and sustainability. With further developments to address current challenges, it can enable next-generation materials that do not force a compromise between engineering excellence and ecological responsibility. In summary, nanocellulose materials, with their unique properties and green nature, are on track to significantly influence the future of materials engineering, enabling innovations that are strong, smart, and sustainable.

## Figures and Tables

**Table 1:** Number of articles published worldwide on bacterial cellulose and plant nanocellulose (2015–2019) [17].

Year	Number of Publications	
	Plant Nanocellulose	Bacterial Cellulose
2015	346	1300
2016	426	1790
2017	705	2440
2018	820	3270
2019	575	5888

## Data Availability

No new data were created or analyzed in this study. Data sharing is not applicable to this article.
